# Health System Impact of Emergency Department-Based Vascular Access Program in Patients with Difficult Intravenous Access

**DOI:** 10.24908/pocusj.v10i01.18274

**Published:** 2025-04-15

**Authors:** Nathan P. Roll, Shilpa Raju, Micah Ownbey, Jamal Jones, Christy Hopkins, Jennifer Cotton

**Affiliations:** 1 Department of Emergency Medicine, University of Utah Health, Salt Lake City, UT, USA

**Keywords:** USG IV placement, difficult intravenous access, POCUS

## Abstract

**Background:**

Ultrasound guided (USG) peripheral intravenous (PIV) access is a vital use of point of care ultrasound (POCUS) that decreases time to access, reduces need for more invasive access, preserves vasculature, and improves patient experience.

**Objectives:**

We describe the impact of an Emergency Department (ED) based vascular access program with a specialized team of paramedics and emergency medical technicians (EMTs). This team is trained in USG PIV access to assist with patients that have difficult intravenous access (DIVA) both in the ED and throughout the academic medical center.

**Methods:**

This descriptive report details the implementation, development, and evolution of a highly skilled vascular access team trained in USG PIV placement at a single academic center. Under the guidance of ultrasound fellowship trained, board-certified Emergency Medicine (EM) ultrasound faculty, ED paramedics and EMTs are provided comprehensive training and oversight of USG PIV placement. Program description, patient selection, and billing capture are described. This report met Institutional Review Board exemption criteria as a descriptive quality improvement project.

**Conclusions:**

This report details the formation and maintenance of a highly skilled vascular access team. The team is comprised of paramedics and EMTs who have been trained in USG PIV placement allowing them to care for patients with DIVA. The evolution of this team has allowed the development of a tiered approach to vascular access and vascular preservation throughout the organization, benefitting both patients and hospital staff.

## Introduction

The utility of ultrasound guided (USG) peripheral intravenous (PIV) access for patients with difficult intravenous access (DIVA) has been described in previous studies [1–7]. USG PIV placement can help decrease time to access and the need for central venous access, midline catheter placement, and peripherally inserted central catheter (PICC) use in patients with DIVA [8,9]. With comprehensive training programs and oversight, this technique can be successfully taught and utilized by both nurses, [10–13] paramedics and emergency medical technicians (EMTs) [14–16].

This report describes the development and sustained excellence of a specialized emergency department (ED) based PIV team, including paramedics, advanced and basic EMTs specializing in both non-USG and USG PIV placement for patients with DIVA. While this team initially operated solely in the ED, the program has expanded to support hospital inpatients, as well as patients in the adjacent outpatient clinics and cancer hospital. Previous reports have detailed the use of this procedure to more broadly support patients with DIVA throughout a healthcare organization [8,17]. However, no previous reports that we know of have described a specialized team of paramedics and EMT's that provide vascular access care institution-wide for patients with DIVA.

## Study Setting

This program was implemented within a major academic medical system, which includes a 57,000-visit academic ED, a 500-bed inpatient medical unit, and a 100-bed cancer hospital.

## Program Description

### Goals

The ED Vascular Access Program concentrates on vascular preservation, staff and patient satisfaction, and exceptional patient-centered care. The program emphasizes minimizing PIV access attempts, reducing infection rates at intravenous sites, and decreasing the need for central lines, midline catheters, and PICC access. This report met Institutional Review Board (IRB) exemption criteria as a descriptive quality improvement project.

### Vascular Access Program Details

Since its initiation in 2005, the Vascular Access Program has evolved into a collaborative effort involving both paramedics and EMTs, under the guidance of ultrasound fellowship trained, board-certified Emergency Medicine (EM) faculty. The USG PIV training was initially created by the ultrasound faculty within EM. At the time of creation, there were no other known vascular access programs that trained paramedics and EMTs in USG PIV access. The initial program focused on paramedic training, however, the program expanded to include senior EMTs, which created a sustainable team training model. Senior paramedics and EMTs train and provide oversight for the performance of USG PIV techniques to newer paramedics and EMTs on the vascular access team. Paramedics and EMT's must have at least one year of clinical experience in the ED prior to being eligible for USG PIV training.

The comprehensive peripheral access training program covers various aspects of obtaining access, including general vein anatomy, deep vein anatomy, vein path and bifurcations, compressibility assessment, and identification of artery and nerve bundles. ED leadership supervises ongoing vascular team member competency. Paramedics and EMTs trained in this technique track intravenous procedures in a centralized department database which is accessed monthly by ED management. New paramedics and EMTs entering the program undergo bedside supervision for a minimum of 20 USG PIVs. Once signed off by a supervisor, the ED vascular access team member maintains privileges by performing a minimum of 10 USG PIVs per month.

The ED vascular access team is paged for intravenous placement requests. The requesting unit provides the vascular access team with the patient demographic information and hospital location. The page is linked to a consult order which is placed in the electronic medical record (EMR) by the care team member requesting assistance with intravenous access.

Per the department's vascular access guideline, the vascular access provider is permitted one attempt without ultrasound. If ultrasound is needed, an appropriate vein is identified. Once the vein is cannulated with ultrasound guidance, the vascular team member ensures adequate blood flow, flushes the line, and assesses for pain and extravasation. Distal veins below the elbow take precedence for access, with antecubital vessels reserved for emergent situations.

**Figure 1.**
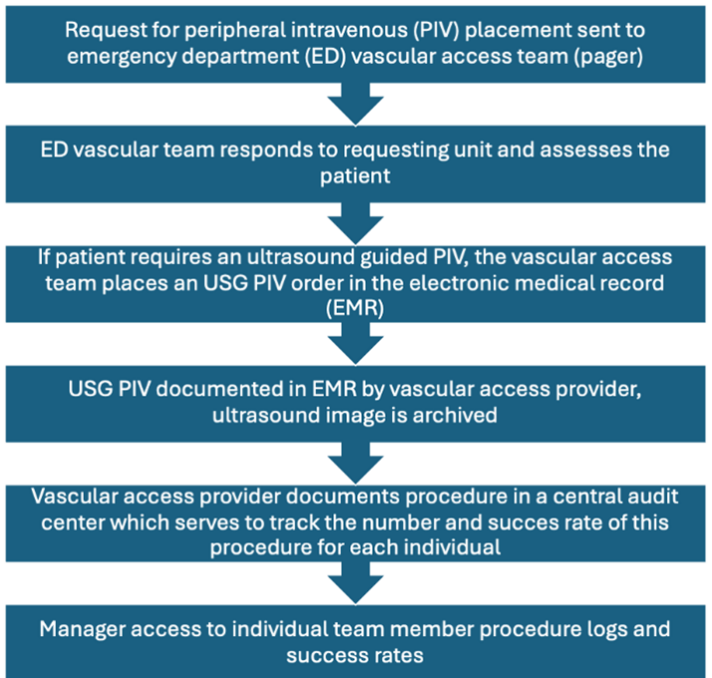
Vascular Access Team Order and Documentation Flow

Documentation for each USG PIV includes the patient's name and date of birth, with ultrasound images confirming the catheter within a vein stored in the Picture Archiving and Communication System (PACS). The images are available for review by EM ultrasound faculty, as needed. (Figure 1)

### Program Growth

Prior to 2016, the vascular access team provided intravenous access services to patients within the system's ED and outpatient areas. In 2016, the vascular access team expanded its services to include inpatient unit coverage at the main hospital campus. In 2019, the team again expanded coverage to include patients with DIVA in the adjacent cancer center.

### Inpatient Selection

The vascular access team is designated as a consulting service within the hospital system for inpatients requiring access for short admissions (1-3 days), surgery, computed tomography (CT) scans, short-term antibiotics, or emergencies. It is important to note that the team refrains from attempting PIV placements in patients with restricted extremity considerations, including the presence of arteriovenous fistulas, history of lymph node removal, prior amputation, infected or fractured limbs, or limb paralysis.

In patients where an appropriate vessel is visualized, the vascular access provider is permitted one attempt without an ultrasound for PIV placement. If an ultrasound is needed, the vascular access provider will place an order in the EMR, and an appropriate vessel will be identified. In cases where no suitable vessels are visualized, the vascular access provider will initiate a consultation with the PICC team which is staffed by trained registered nurses. USG PIVs are preferentially placed in forearm vessels to preserve more proximal vasculature for potential PICC or midline access.

In contrast to the ED vascular access team, the PICC team is primarily consulted for patients requiring long-term antibiotics, individuals with a history of chronic kidney disease, patients with a history of upper extremity thrombosis, patients meeting restricted extremity criteria, or patients with a history of complications with PIVs such as phlebitis, thrombosis, and infiltrations. This strategic approach ensures the optimal utilization of both teams based on their expertise and the specific needs of the patients they serve.

## Billing

### Billing Documentation

**Figure 2.**
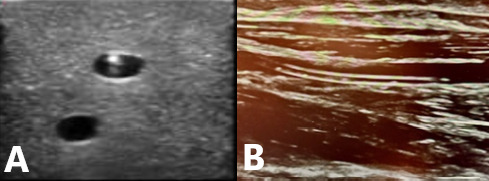
Peripheral intravenous cannula within the lumen of the vein. A is a short axis view of the cannula (in simulation gel) and B is a long view of the cannula.

Documentation for USG PIVs includes the following elements: date and time, location, size and length of the catheter, technique (USG), two indications for the use of USG PIV, and arm circumference. The vascular access team archives images showing an intraluminal catheter and provides a post-intravenous assessment, ensuring the charge is entered into the records (Figure 2). All PIV attempts are documented in the EMR, even if unsuccessful.

Initially, billing for complex access procedures was executed manually through a paper-based system. However, with the adoption of EMR, the vascular access team implemented a specific DIVA order set in 2019. The implementation of an electronic order replaced the conventional manual billing methods. This improved the charge capture rates and the accuracy and efficiency of billing for advanced intravenous access procedures.

### Emergency Department Peripheral Intravenous Procedure Volumes and Estimated Payments

Prior to 2016, the Vascular Access Program relied on manual documentation of USG PIV placements. Between 2013 and 2015 an average of 785 USG PIV procedures per year were billed for, resulting in an annual estimated revenue of approximately $140,000. In 2016, with the adoption of a new EMR, the vascular access team began electronically entering orders for USG PIV placements, which led to a notable increase in annual USG PIV charge capture. Between 2016 and 2018, the vascular access team billed for an average of 2,865 USG PIVs per year, with an estimated annual revenue of over $675,000.

In 2019, a specialized USG PIV (DIVA) order set was implemented for the vascular access team. The DIVA order set dramatically increased charge capture, increasing the average USG PIV procedures in the ED to over 11,215 USG PIV annually. With an average ED census of 57,000 patients, USG PIV orders are initiated on approximately 20% of the total ED population. Estimated revenue from this procedure increased to over $2.4 million dollars with the implementation of a DIVA specific order set (Table 1). Revenue estimates are based on the institutional average reimbursement rate of 41% of charges. This procedure uses the facility charge code of “Ultrasound for Vascular Access” (hospital billing code).

**Table 1.** Emergency Department Charges and Estimated Revenue from Medic Ultrasound Guided (USG) Peripheral Intravenous (PIV) Program

	2013-2015	2016-2018	2019-2023
Average Number of USG PIV billed	785	2865	11,215
Average Annual Charges Billed	$ 339,673	$ 1,647,403	$ 6,014,465
Estimated Annual Revenue*	$ 139,266	$ 675,435	$ 2,465,931

* 41% of Charges

As of January 1, 2024, the Vascular Access Program supported over 18 full-time equivalent providers, with a total annual salary and benefits cost of $1,241,000. Crucially, the program's financial sustainability is underscored by its ability to offset these costs through improved charge capture and increased annual revenue, exemplifying its contribution to the overall fiscal health of the healthcare system.

### Inpatient Ultrasound Guided Peripheral Intravenous Placement Volumes

Between January 1 and December 31, 2023, the vascular access team was consulted 11,867 times for intravenous access by inpatient services. While the majority of the inpatient consults had intravenous access secured without need for ultrasound guidance, a total of 2,058 (17.3%) patients did require USG PIV placement. Reimbursement for inpatient USG PIV placement is bundled into the inpatient charges, and are not billed separately by the vascular access team.

## Program Impact

This report presents a detailed overview of a self-sustaining vascular access program designed to provide 24-hour support within the organization, catering specifically to patients with DIVA. Central to this program's success is the formation of a highly specialized team following a thoughtful strategy, which ensures that vascular access providers receive standardized training, ongoing oversight of procedural skills, and extensive experience with specialized patient populations. This specialized team works to support nurses throughout the institution and helps to foster a culture of exceptional patient care and vascular preservation. The tiered PIV placement guidelines allow for appropriate escalation to more advanced access options as dictated by each patient's unique needs. This adaptive approach enhances the overall quality of care provided to individuals with challenging intravenous access.

## Limitations

There are several important limitations of this report. This report is a descriptive study without statistical analysis and direct comparisons to other existing studies in the literature. The success of the USG vascular access team is the result of continuous process improvement, collaboration between EM ultrasound faculty and the vascular access team, a dedicated focus on training and quality improvement, and exceptional leadership. Transferability to other institutions would depend heavily on resources, staffing, and leadership at individual sites.

Tracking and billing of total intravenous starts and USG PIV placements is more robust after the introduction of vascular access team consult orders and the initiation of a DIVA order set. Despite the addition of a specific order set for this procedure, actual PIV placements may be under-represented given that the adoption of the consult order and order set is not universal. As well, the impact of improving ultrasound technology on the likelihood of success of USG PIV placement was not examined.

The reported reimbursement rates are based on estimates derived from the current payor mix and average reimbursement rates within one academic center. The wide variability in payor mixes and reimbursement rates across different healthcare institutions limits the broad applicability of our findings to other organizations. Each healthcare system has its unique financial landscape. As such, caution should be exercised when attempting to extrapolate our results to institutions with different contextual factors.

## Conclusion

This report details the successful implementation of a specialized ED vascular access team comprised of paramedics and EMTs with focused training in USG PIV placement for patients with DIVA. The team not only cares for patients in the ED with DIVA, but is now an integral part of patient care throughout the institution. The team has enabled a tiered approach to vascular access and vascular preservation throughout the organization, benefitting both patients and hospital staff. The team's financial self-sustainability is a noteworthy achievement facilitated by the implementation of improved charge capture methodologies. Not only does this contribute to the team's autonomy, but it also helps to augment the institution's overall operating margin. The ongoing dedication to exceptional patient care, focus on continuous improvement, and strong collaborative efforts will continue to support this highly specialized team moving forward.
